# Supplementary material for: Similarities and Differences in Extracellular Vesicle Profiles between Ischaemic Stroke and Myocardial Infarction
Source: Biomedicines. 2020 Dec 24;9(1):8. doi: 10.3390/biomedicines9010008 (PMC7824002; doi:10.3390/biomedicines9010008)
Supplement: Supplementary file 1 [file biomedicines-09-00008-s001.pdf]

biomedicines

MDPI

Supplementary Materials

Table S1. Proteins identified in EVs from HC, MI and IS that have not been found in vesiclepedia.

Proteins	HC	MI	IS
Immunoglobulin mu heavy chain	Identified	Identified	Identified
Keratin, type II cytoskeletal 1	Identified	Identified	Identified
Immunoglobulin gamma-1 heavy chain	Identified	Identified	Identified
Haptoglobin-related protein	Identified	Identified	Identified
Apolipoprotein A-IV	Identified	Identified	Identified
Immunoglobulin kappa light chain	Identified	Identified	Identified
Alpha-1-antitrypsin	Identified	Identified	Identified
Complement factor H	Identified	Identified	Identified
Prothrombin	Identified	Identified	Identified
Immunoglobulin alpha-2 heavy chain	Identified	Identified	Identified
Immunoglobulin heavy variable 3-30	Identified	Identified	Identified
Serotransferrin	Identified	Identified	Identified
Antithrombin-III	Identified	Identified	Identified
Immunoglobulin J chain	Identified	Identified	Identified
Immunoglobulin heavy variable 3-72	Identified	Not Identified	Identified
Immunoglobulin heavy variable 5-51	Identified	Identified	Identified
Immunoglobulin heavy variable 1-46	Identified	Not identified	Identified
Immunoglobulin heavy variable 3-15	Identified	Not identified	Identified
Immunoglobulin heavy variable 3-73	Identified	Not identified	Not identified
Plasma protease C1 inhibitor	Identified	Identified	Identified
Hemoglobin subunit alpha	Identified	Identified	Identified
Immunoglobulin heavy variable 6-1	Identified	Identified	Identified
Complement component C6	Identified	Identified	Identified
Immunoglobulin heavy variable 4-4	Identified	Not identified	Identified
Immunoglobulin heavy variable 3-64D	Identified	Identified	Identified
Complement C1q subcomponent subunit B	Identified	Not identified	Identified
Immunoglobulin lambda variable 3-10	Identified	Identified	Identified
Immunoglobulin kappa variable 3-20	Identified	Identified	Identified
Immunoglobulin lambda variable 8-61	Identified	Identified	Identified
Immunoglobulin kappa variable 3D-11	Identified	Identified	Identified
Immunoglobulin lambda variable 1-51	Identified	Identified	Identified
Immunoglobulin lambda variable 3-19	Identified	Identified	Identified
Immunoglobulin delta heavy chain	Identified	Not identified	Identified
Immunoglobulin lambda variable 7-43	Identified	Identified	Identified
Immunoglobulin heavy variable 1-2	Identified	Not identified	Identified
Lysozyme C	Identified	Not identified	Identified
Cathepsin D	Identified	Not identified	Identified
Immunoglobulin lambda variable 3-9	Not identified	Not identified	Identified
Immunoglobulin heavy variable 4-28	Not identified	Not identified	Identified
Immunoglobulin lambda variable 1-47	Not identified	Not identified	Identified
Complement C1q subcomponent subunit A	Not identified	Not identified	Identified
Immunoglobulin heavy variable 4-34	Not identified	Not identified	Identified
Filaggrin	Not identified	Not identified	Identified
Skin-specific protein 32	Not identified	Identified	Identified
